# A comprehensive study of the hormetic influence of biosynthesized AgNPs on regenerating rice calli of indica cv. IR64

**DOI:** 10.1038/s41598-019-45214-y

**Published:** 2019-06-19

**Authors:** Markandan Manickavasagam, Gadamchetty Pavan, Venkatachalam Vasudevan

**Affiliations:** Department of Biotechnology, Bharathidasan University, Tiruchirappalli-620 024, Tamil Nadu, India

**Keywords:** Agricultural genetics, Agricultural genetics, Agricultural genetics, Agricultural genetics

## Abstract

Rice is one of the most widely cultivated crops worldwide; however, it is not amenable to genetic manipulations, owing to its poor response to tissue culture and regeneration *in vitro*. To improve its response to tissue culture, we evaluated the influence of biosynthesized silver nanoparticles on callus induction, regeneration and rhizogenesis in Indica rice cv. IR64. Silver nanoparticles were biosynthesized by using silver nitrate and *Parthenium hysterophorus* plant extract, and were characterized by UV-visible spectroscopy, Fourier-transform infrared spectroscopy, Transmission electron microscopy and X-ray diffraction. The biosynthesized silver nanoparticles (PHAgNPs), when supplemented in tissue culture medium, promoted callus induction frequency, callus regeneration and rhizogenesis at concentrations of 10 mg l ^−1^, 5 mg l^−1^ and 10 mg l^−1^, respectively. Further examination of the endogenous hormonal levels in regenerating calli revealed that AgNPs enhanced regeneration by alleviating abscisic acid and ethylene levels in the plant tissue. The stimulatory influence eliciting the regeneration response was found to be optimal with the supplementation of 5 mg l^−1^ PHAgNPs in the regeneration medium; the malondialdehyde, proline and hydrogen peroxide levels were also lower than those in the control, thus suggesting improved antioxidant status. Our results indicated that biosynthesized PHAgNPs may have the potential to positively influence tissue culture of recalcitrant varieties.

## Introduction

Indica Rice is prominently cultivated in the tropical and subtropical regions of Asia, and it accounts for 80% of the rice cultivated worldwide. With the increasing demand for produce, there is a need to improve the tolerance of germplasm to biotic and abiotic stress conditions without compromising yield^1,2^.

Genetic transformation of rice calli with *Agrobacterium tumefaciens* is frequently used to improve the crop, because it ensures a low copy number and stable integration of T-DNA. However, *Agrobacterium* mediated transformation of indica rice calli has limitations, owing to poor regeneration and callogenesis, which are influenced by numerous internal and external factors^3^. With the advent of nanobiotechnology, researchers have demonstrated that application of nanotechnology in plant tissue culture has shown promise, positively influencing the germination rate of seeds, plant growth, metabolite production, organogenesis, callus induction frequency and regeneration frequency, as well as eliminating microbial contamination^4^.

Plant growth and development are modulated by endogenous plant growth regulators (PGRs). Amongst all the identified PGRs, auxins, cytokinins, gibberellins, abscisic acid (ABA) and ethylene are the most prominent natural plant hormones. Auxin is the most important modulator found throughout the plant, and its accumulation is imperative for initiation of apical meristem. Cytokinin is involved in germination, meristematic functions and leaf senescence. The interaction of auxin and cytokinin is crucial for the development of plants, and consequently these hormones are commonly employed in *in vitro* plant tissue culture to regulate differentiation in explants. Gibberelins are involved in growth, organ elongation, seed development and regulation of flowering time. ABA is regulated by external factors involved in stomatal closure, germination, root elongation and flowering, and it is part of a complex regulatory network that includes auxins and cytokinins and is imperative for embryogenesis and shoot regeneration. Another important PGR is ethylene, a gaseous hormone that primarily influences ripening of fruits and senescence in plants^5,6^. Studies have demonstrated that *in vitro* tissue culture results in accumulation of ethylene, and supplementation with silver nanoparticles (AgNPs) in plant tissue culture medium makes explants healthier and improves their growth vigour and regeneration frequency, effects attributable to the ability of silver ions to inhibit ethylene synthesis^7–11^. However, the influence of AgNPs on other PGRs during regeneration remains unclear.

Another study has reported that plant tissues, even when propagated under optimal conditions, produce reactive oxygen species (ROS), an unavoidable by-product of general plant metabolism that is detrimental for plant growth and development. Even though the harmful free radicals are reduced by an internal antioxidant system, the process consumes vital resources in the cells, thus hindering growth and development^12^. Silver ions, beyond functioning as ethylene inhibitors, serve as electron acceptors and donors in redox reactions, especially in supporting exchange of electrons with CO_3_^+^ and Fe_2_^+^ ^13^, thereby reducing the ROS and alleviating the strain on the plant antioxidant system. However, compared with silver ions, AgNPs are more efficient in chemical reactions and interact better with the surrounding environment because of their higher surface area to mass ratio^7^. Hence, in the present study, we attempted to correlate the influence of exogenously supplemented AgNPs on endogenous ROS as well as PGR levels in regenerating calli, to better understand the influence of AgNPs on plant development. AgNPs, because of their small size (1–100 nm) possess unique optical and physiochemical properties, and thus are used in various fields for conduction, biological detection, catalysis, wound healing, anti-microbial activity and phytostimulation^14^.

Synthesis of AgNPs through chemical and physical methods requires toxic chemicals and complex purification steps. However, AgNPs can be synthesized by utilizing plant extracts, a process that is simple, economical and eco-friendly. *Parthenium hysterophorus* (PH), used for bio-fabrication of AgNPs in the present study, is one of the most difficult weeds to control worldwide. It is toxic to animals and harmful to biodiversity, and it is responsible for economic losses in agriculture. Control measures such as burning the weeds, spraying chemical herbicides, and introducing pests, mycoherbicides, and competitive crops each have their own constraints. However, utilizing PH plants for biosynthesis of nanoparticles (NPs) is a way to put these weeds to good use^15,16^.

In the present study, we used a bio friendly route to synthesize NPs by using PH plant extract, an environmentally benign process resulting in more stable and biocompatible NPs, as compared with the chemical reduction process, wherein harsh chemicals adsorb onto the surfaces of NPs and render them toxic^17^. The biosynthesized silver NPs positively influenced callogenesis, regeneration and rhizogenesis in commonly grown Indica rice cv. IR64 plants *in vitro*. Because plant hormones play a crucial role in the sensing ability and adaptability of plants^18^, we correlated the hormonal profile with the regeneration efficiency of the calli exposed to PHAgNPs. The PHAgNPs were found to enhance regeneration efficiency by decreasing ethylene, ABA and ROS levels in the regenerating calli.

## Results

### Synthesis and characterization of PHAgNPs

**Figure 1:**
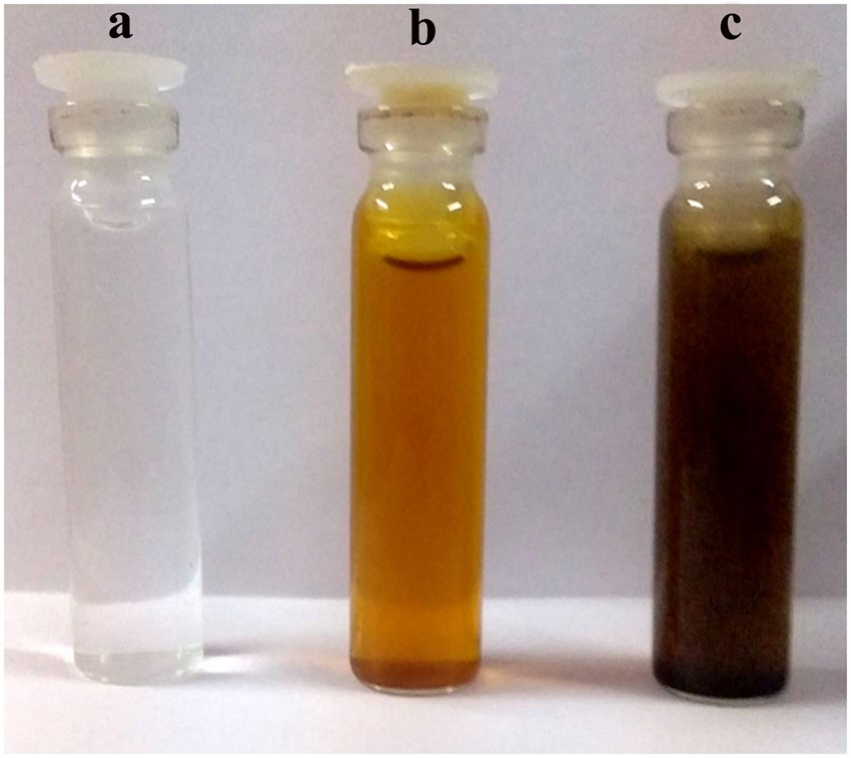
Biosynthesis of PHAgNPs. (**a**) AgNO_3_ solution (1 mM). (**b**) Parthenium plant extract. (**c**) plant extract with 1 mM AgNO_3_ after incubation for 30 min at 60 °C.

**Figure 2:**
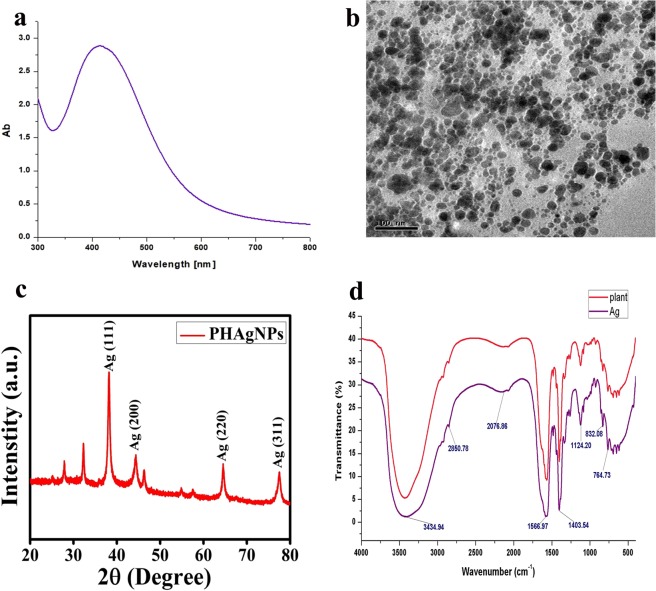
Characterization of silver nanoparticles. (**a**) UV–VIS spectra of biosynthesized PHAgNPs. (**b**) TEM image of PHAgNPs. (**c**) XRD analysis of PHAgNPs. (**d**) FTIR spectrum analysis of PHAgNPs and plant extract.

The biosynthesis of PHAgNPs was initially confirmed visually, as the reaction mixture of 1 mM AgNO_3_ and plant extract changed colour from pale yellow to deep brown following incubation (Fig. 1). The colour change was attributed to surface plasmon resonance caused by collective oscillation of free conduction electrons^14^. When subjected to UV-vis spectroscopic analysis, the PHAgNPs produced an absorption peak at 420 nm, thereby confirming the stability of synthesized PHAgNPs (Fig. 2a).

Transmission electron microscopy (TEM) indicated that the PHAgNPs were mostly spherical in shape and poly dispersed, with sizes ranging from 1 to 50 nm (Fig. 2b).

Fourier-transform infrared spectroscopy (FTIR) spectroscopy was carried out to identify the functional groups from PH involved in the reduction of Ag^+^ and capping of silver NPs. The spectrum bands were observed at 3434.94 cm^−1^, 2850.78 cm^−1^, 2076.86 cm^−1^, 1403.54 cm^−1^, 1124.20 cm^−1^, 832.08 cm^−1^, 764.73 cm^−1^ and 566.97 cm^−1^, corresponding to O–H stretching in an alcohol, C–H stretching in an alkane, N=C=S stretching in isothiocyanate, O–H bending in carboxylic acid, C–O stretching in an aliphatic ether, C–C bending in an alkene, C–H stretching and C–I stretching in a halo compound, respectively. The results confirmed the involvement of plant extract in the bio-reduction of Ag^+^ ions and encapsulation of Ag NPs (Fig. 2d).

X-ray diffraction (XRD) was used to characterize the crystalline nature of the PHAgNPs. After reduction, well resolved Bragg reflections for PHAgNPs were obtained at 2θ = 38.2°, 44.3°, 64.4° and 77.5°, corresponding to the crystal lattice planes [111], [200], [220] and [311] of the face centred cubic (fcc) structure of silver (JCPDS files No. 89–3722). The Bragg reflections at 2θ  =  27.8° and 32.3° might represent the bioorganic phase on the surfaces of the AgNPs (Fig. 2c). The average size of NPs was determined to be ~25 nm with Debye–Scherrer’s equation.

### Effects of AgNPs on callus induction, regeneration and rooting *in vitro*

**Figure 3:**
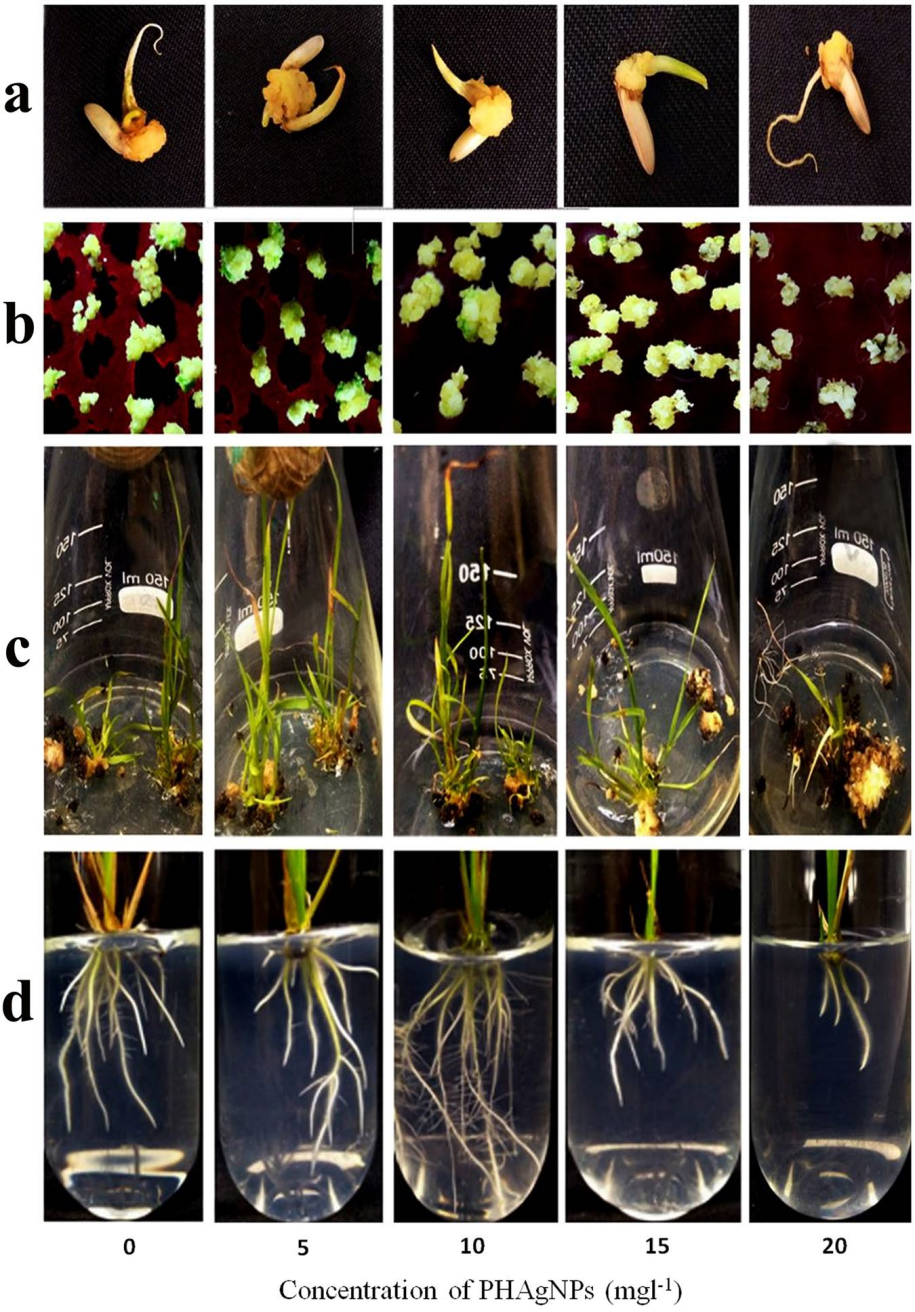
Effect of PHAgNPs (0, 5, 10, 15 and 20 mg l^−1^) on callogenesis (**a**), regeneration (**b**,**c**) and rooting (**d**) of *Oryza sativa* L. cv. IR64.

The embryogenic calli generated from mature seeds displayed a variable response towards different concentrations of PHAgNPs in the callus induction medium (Fig. 3a).

The callus induction frequencies of rice with respect to different concentrations of PHAgNPs are given in Table 1. The callus induction response was highest with PHAgNP concentration of 10 mg l^−1^ with callus induction frequency of 82%, followed by 69% with 5 mg l^−1^ PHAgNPs, 37% with 15 mg l^−1^ PHAgNPs and 16% with 20 mg l^−1^ PHAgNPs, relative to control medium devoid of PHAgNPs, which displayed a callus induction frequency of 62% (Table 1). At lower concentrations of PHAgNPs, the calli had a friable texture and creamy appearance; however, with increasing concentrations of PHAgNPs, the calli turned brown.

**Table 1:** Response of tissue cultured rice to different concentrations of PHAgNPS.

	0	5	10	15	20
Callus induction frequency (%)	62 ± 5.2b	69.4 ± 6b	82.4 ± 5.2a	37 ± 4.7c	16.6 ± 4.5d
Regeneration frequency (%)	41.1 ± 4.2b	61 ± 6.3a	40 ± 4.7b	18.9 ± 8.3c	4.4 ± 1.6d
Root length (cm)	3.4 ± 0.3bc	4 ± 0.2b	4.9 ± 0.3a	2.7 ± 0.3c	1.16 ± 0.4d
Number of roots	9.7 ± 0.2bc	10 ± 0.3b	11.2 ± 0.6a	9 ± 0.5c	4.8 ± 0.2d

Influence of PHAgNPs on callus Induction frequency, regeneration frequency, root development and root length.

Means ± Standard Deviation, n = 3. Means followed by same letter for columns (E.g. a, b, bc) are not significantly different at p ≤ 0.05 as determined by DMRT. (0- Control medium, 5 – Medium with 5 mg l^−1^ PHAgNPs, 10–10 mg l^−1^ PHAgNPs, 15–15 mg l^−1^ PHAgNPs, 20–20 mg l^−1^ PHAgNPs).

The induced calli from IR64 seed explants were shifted to regeneration medium with different concentrations of PHAgNPs and control medium. Green spots formed on the calli within 14 days (Fig. 3b). The green calli were further sub-cultured onto fresh medium, and the regeneration potential was documented (Table 1). The regeneration efficiency was observed to be highest (61%) on culture medium supplemented with 5 mg l^−1^ PHAgNPs, in contrast to the optimal PHAgNP concentration required for efficient callus induction. The increase in PHAgNP concentration decreased the regeneration frequency. At PHAgNP concentrations of 10 mg l^−1^, 15 mg l^−1^ and 20 mg l^−1^, the regeneration frequency was 40%, 18% and 4%, respectively, relative to the regeneration frequency (41%) observed on control medium (Table 1).

The individual regenerated plants were transferred to rooting medium. After 2 weeks, application of NPs up to only a certain concentration increased the number of roots and the root length. The greatest rooting response with healthy secondary roots and root hair was observed at a concentration of 10 mg l^−1^, followed by 5 mg l^−1^ and 15 mg l^−1^. At a PHAgNP concentration of 20 mg l^−1^, the root length and number were drastically lower than those observed with control medium (Fig. 3d, Table 1).

### Changes in expression of PGR genes

**Figure 4:**
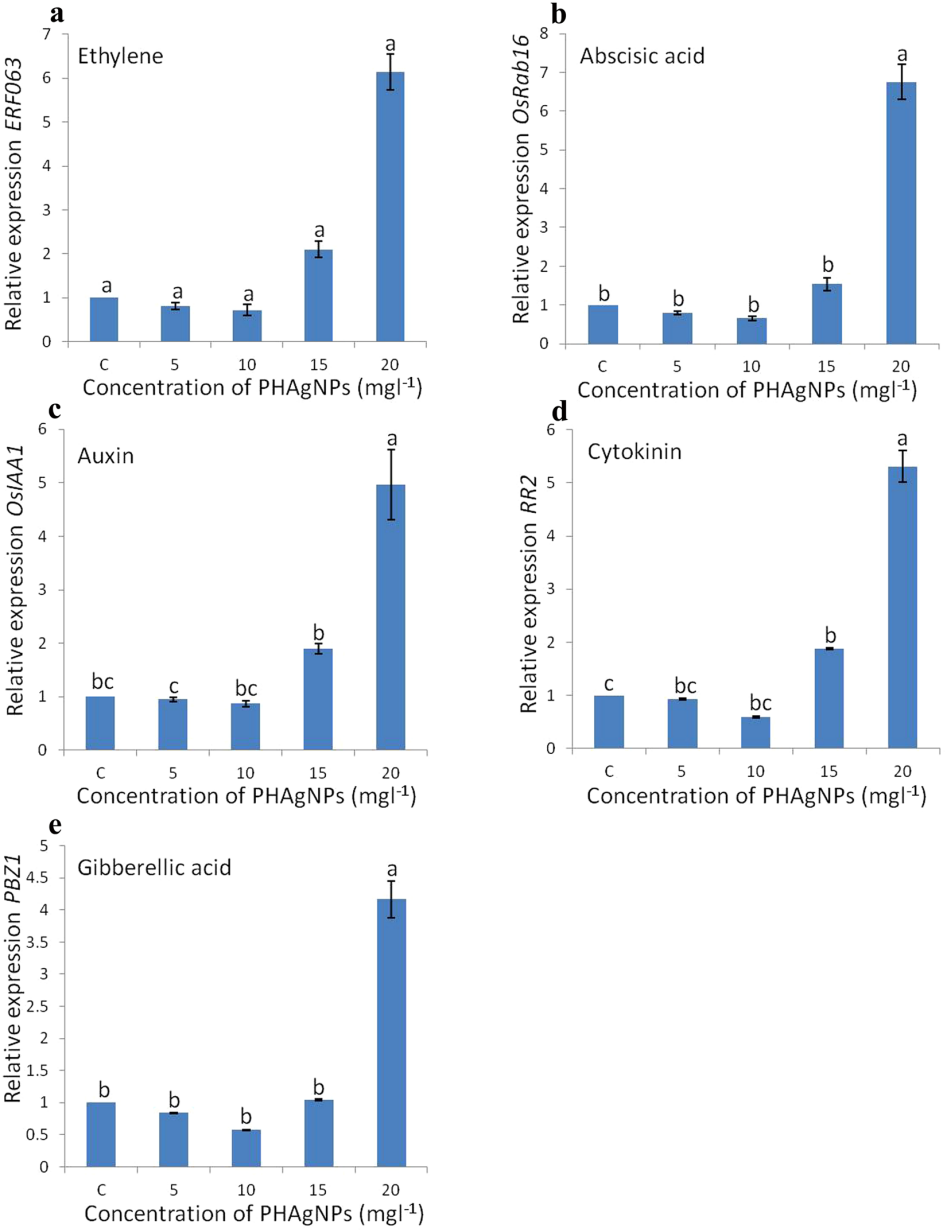
qRT-PCR quantification of mRNA levels of Plant Growth Regulator responsive genes in regenerating callus of rice. (**a**) *ERF063* ethylene responsive gene. (**b**) *OsRab16* abscisic acid responsive gene. (**c**) *OsIAA1* auxin responsive gene. (**d**) *RR2* cytokinin responsive gene. (**e**) *PBZ1* gibberellic acid responsive gene. Ubiquitin level was used as a reference. Data are mean ± standard error (n = 3). Means followed by same letter for columns (E.g. a, b, bc) are not significantly different at p ≤ 0.05 as determined by DMRT.

Exposure of calli to PHAgNPs significantly influenced the expression of PGR responsive genes. The basic PGR responsive genes were downregulated upon exposure to PHAgNPs (5 mg l^−1^ and 10 mg l^−1^) as compared with control treatment, and the lowest expression was at 10 mg l^−1^. The transcript levels of ethylene, ABA, auxin, cytokinin and gibberellic acid responsive genes decreased by a maximum of 0.3, 0.35, 0.2, 0.3 and 0.4 fold, respectively, after culture in medium with 10 mg l^−1^ PHAgNPs. However above 10 mg l^−1^, the expression of all the genes markedly increased, probably because of the stress induced by increased exposure to heavy metal (Fig. 4).

### Proline, H_2_O_2_ and malondialdehyde levels

**Figure 5:**
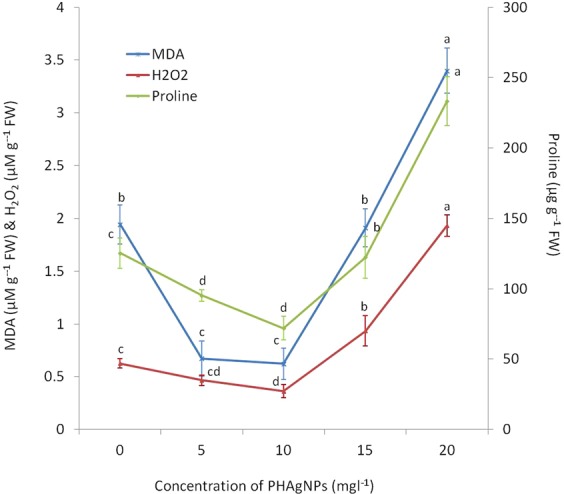
MDA, H_2_O_2_ and Proline content of regenerating calli, in response to different concentrations of supplemented PHAgNPs. Data are mean ± standard deviation (n = 3). Means followed by same letter for columns (E.g. a, b, bc) are not significantly different at p ≤ 0.05 as determined by DMRT.

Under control conditions, the proline, H_2_O_2_ and malondialdehyde (MDA) levels were 1.94 µM g^−1^ FW, 0.63 µM g^−1^ FW and 125.36 µg g^−1^ FW, respectively, and the values significantly decreased when the explants were cultured on medium supplemented with 5 mg l^−1^ and 10 mg l^−1^ PHAgNPs. The lowest levels were recorded on medium fortified with 10 mg l^−1^ PHAgNPs, with 0.62 µM g^−1^ FW, 0.36 µM g^−1^ FW and 72 µg g^−1^ FW for proline, H_2_O_2_ and MDA respectively. However, the H_2_O_2_, proline and MDA levels sharply increased when the explants were exposed to 15 mg l^−1^ and 20 mg l^−1^ PHAgNP concentrations (Fig. 5).

## Discussion

In the present study, the biosynthesized AgNPs were validated with FTIR, which analysed the displacement in the absorbance rate of biomolecules in PHAgNPs compared with that of biomolecules in PH plant extract. FTIR analysis confirmed the involvement of alcohol, alkane, isothiocyanate, carboxylic acid, aliphatic ether and alkene groups in the biosynthesis of AgNPs. Similarly, earlier reports have suggested that the biosynthesis involves the reduction and capping of AgNPs by saponin, free amino acids, galactose and other compounds present in the PH leaf extract^19–21^. The XRD pattern of PHAgNPs was determined by using JCPDS intensities (JCPDS files No. 893722), which suggested that the biosynthesized NPs have a face centred cubic structure, and the biosynthesized NPs have an average size of ~25 nm and a mostly spherical shape, as evidenced by TEM and calculations with Debye–Scherrer’s equation. The results confirmed the synthesis of the PHAgNPs and were consistent with the previously reported results for phase centred cubic AgNPs^22,23^.

Plant tissue culture has become an indispensable tool for commercial micropropagation of plants, genetic transformation for crop improvement and functional genomics studies. In the present study, the application of biosynthesized PHAgNPs increased callus induction in a dose dependent manner, and the greatest response was found with MS medium supplemented with 2.5 mg l^−1^ 2,4-D and 10 mg l^−1^ PHAgNPs. A 20% increase in callus induction frequency was observed with medium containing 10 mg l^−1^ PHAgNPs compared with control MS medium. However, further increases in PHAgNP concentration decreased the rate of callogenesis, and 15 mg l^−1^was a sub-optimal concentration. At a 20 mg l^−1^ concentration of NPs, the induction response decreased further, by 46% as compared with control medium. These results contrast with those from previous experiments^24,25,26^ in which treatment with AgNPs increased the callus induction frequency and fresh weight of *S. nigrum* in a dose and time dependent fashion. However, ZnO and TiO2 NPs at concentrations of 5 mg l^−1^ and 20 mg l^−1^, respectively, resulted in the highest induction of calli from indica rice cv. RD49^27^. After transfer of the induced calli to regeneration medium, the effects of PHAgNPs differed. Control medium resulted in a regeneration frequency of 41%, whereas the highest greening of calli and regeneration frequency (61%) was observed with CIM supplemented with PHAgNPs at a concentration of 5 mg l^−1^. However, greening and organogenesis drastically declined with increasing concentrations of NPs in the culture medium. Similarly, a previous study has reported that TiO_2_NPs (20 mg l^−1^) resulted in the highest regeneration percentage of rice calli; however, any further increase in concentration of NPs resulted in a decline in the regeneration frequency^27^. A further investigation into the influence of PHAgNPs on the expression of PGR responsive genes encoding five important plant growth hormones—auxin, cytokinin, gibberellic acid, ethylene and ABA^28^—revealed that at 5 mg l^−1^ PHAgNP concentration, the auxin and cytokinin expression levels were not significantly altered, whereas the ethylene and ABA responsive gene expression decreased, and the regeneration frequency in calli was enhanced. When 10 mg l^−1^ PHAgNPs were supplemented in the regeneration medium, a further decline in ABA and ethylene hormonal levels was observed, which in turn should have increased the regeneration frequency. However, cytokinin responsive gene expression levels also further declined relative to auxin levels, thus disturbing the cytokinin:auxin ratio, which probably was responsible for the decrease in regeneration frequency. The gibberellic acid responsive gene expression levels remained in concurrence with cytokinin and auxin responsive gene levels, thus suggesting little to no influence on enhancing the regeneration of induced rice calli. In agreement with our results, previous reports have suggested that a balance in cytokinin and auxin ratio is crucial for regeneration response in undifferentiated tissues^29^. In rice, the auxin and cytokinin ratio has been demonstrated to determine regeneration from calli, and an increase in the supplemented auxin to cytokinin ratio suppresses plant regeneration frequency in calli^30^. Furthermore, at a PHAgNP concentration of 20 mg l^−1^ a substantial (>6 fold) increase in ethylene and ABA concentrations was observed, which was detrimental for regeneration from calli (Fig. 4). An increased ABA level in plants is an indicator of environmental stress and is detrimental to growth and development^6^. Another mechanism through which AgNPs influence regeneration is that when explants are cultured in closed containers (*in vitro*) their development is severely influenced by plant-produced gases such as ethylene, which even in trace amounts can regulate senescence in plants^7^. Silver ions or nano silver suppresses the influence of ethylene by acting as an ethylene perception inhibitor, by down regulating the genes involved in ethylene perception, as reported previously in *Arabidopsis thaliana*^31^. Finally, when the regenerated shoots were subjected to rooting *in vitro*, they displayed more roots per plant and longer root length when grown on culture medium augmented with 10 mg l^−1^ PHAgNPs. At concentrations above and below 10 mg l^−1^, a steady decline in root generation was observed, thus suggesting a hormetic influence of PHAgNPs. Similarly, in *Arabidopsis*, supplementation with AgNPs in the rooting medium positively influences root growth^31^. A previous study has also reported a positive influence of AgNPs on plant root growth through upregulation of auxin synthesis^32^.

Further analysis of the antioxidant status of the regenerating calli revealed that MDA, proline and H_2_O_2_ levels decreased in calli cultured on medium supplemented with 5 mg l^−1^ and 10 mg l^−1^ PHAgNPs (Fig. 5), and an increase in MDA and H_2_O_2_ levels was observed in calli cultured on medium supplemented with 15 and 20 mg l^−1^ PHAgNPs, an effect correlating with the decrease in regeneration efficiency of the calli. Metal NPs reportedly induce systemic stress, elevating ROS and expression of genes involved in DNA repair, in a manner strongly dependent on the exposed concentration of NPs. However, at lower concentrations, NPs have a negligible or positive influence on plant growth^18^. Similarly, in *Brassica* seedlings, treatment with AgNPs induces antioxidant enzymes, thereby decreasing the hydrogen peroxide (H_2_O_2_), MDA and proline levels, thus suggesting an improvement in the antioxidant status of the plant^13^. ROS damages proteins, lipids and nucleic acids, thereby leading to the development of MDA, which is toxic to plants^33^. Hence, a decrease in MDA, H_2_O_2_ and proline levels implies a decrease in ROS production and consequently stress levels in the plant tissues, as regulated by silver ions^13^.Thus, we conclude that AgNPs influence organogenesis, tissue growth and development through multiple mechanisms.

Supporting our data, earlier studies have also reported a hormetic influence of AgNPs, CuNPs and ZnONPs on regeneration in *Vanilla planifolia*, rice and banana, respectively^34,17,35^. In *Tecomella undulata*, supplementation with AgNPs (10 mg l^−1^) positively influences callus formation, shoot induction and the number of generated shoots from explants^11^.

Apart from AgNPs, other prominent NPs used in tissue culture are aluminium oxide (Al_2_O_3_), CuO, iron oxide (Fe_3_O_4_), gold (Au), magnesium oxide (MgO), nickel (Ni), silicon (Si), SiO_2_, titanium dioxide (TiO_2_) and ZnO^4^. Previous reports in rice tissue culture have demonstrated that supplementation with ZnO (5 mg l^−1^) and TiO_2_ (20 mg l^−1^) NPs enhances callus induction by 2% and 6% in indica rice cv. RD49. Further supplementation with TiO_2_ NPs (20 mg l^−1^) in the regeneration medium enhances the callus regeneration frequency by approximately 8% relative to the control; however ZnO NPs, compared with control treatment, do not significantly enhance plant regeneration^27^. Another study has demonstrated that supplementation with TiO_2_ (50 mg l^−1^) enhances callus induction frequency by 4% and regeneration frequency by approximately 2% in *Oryza sativa* L. cv. Suphanburi1 and Suphanburi90^36^. Similarly supplementation of rice tissue culture medium with 5 mg l^−1^ and 20 mg l^−1^ nanocarbon enhances the frequency of callus induction (by 1%) and regeneration (by 3%), respectively, as compared with growth on control medium devoid of nanocarbon in *Oryza sativa* L. cv. Khao Dawk Mali 105^37^. Anwaar *et al*.^17^ have reported that supplementation with CuONPs (10 mg l^−1^) in callus induction medium enhances callus induction frequency by 32%, 44%, 28% and 36% in rice varieties Basmati 2000, Basmati 370, Basmati 385 and Super basmati, respectively. Furthermore, supplementation with 20 mg l^−1^, 20 mg l^−1^, 20 mg l^−1^ and 15 mg l^−1^ CuONPs enhances regeneration frequency by 38% (Basmati 2000), 22% (Basmati 270), 32% (Basmati 385) and 28% (Super Basmati). From the above reports, we conclude that non-silver NPs of any origin have clear potential to enhance tissue culture of recalcitrant crop varieties, a possibility remaining to be explored further.

We report that PH biosynthesized AgNPs, when supplemented in tissue culture medium, prominently enhance callus induction frequency, regeneration frequency and rhizogenesis at concentrations of 10 mg l^−1^, 5 mg l^−1^ and 10 mg l^−1^, respectively, in the recalcitrant indica rice cv. IR64. The above study sheds light on the endogenous hormonal and ROS levels corresponding to the hormetic influence of AgNPs on shoot regeneration from induced rice calli. We therefore conclude that biosynthesized AgNPs have enormous potential in refining tissue culture of recalcitrant crops. In addition, elucidating the activity of AgNPs on endogenous ROS and phytohormone levels may provide a lucrative platform for crop manipulation to enhance growth and yield.

## Materials and Methods

The phytofabrication and characterization of AgNPs was carried out as previously described with several modifications^38^.

### Preparation of *Parthenium hysterophorus* aqueous extract

Fresh disease-free leaves of locally collected PH were rinsed with distilled water and shadow dried for 5–7 days to remove residual moisture. The dry leaves were crushed to a fine powder with a kitchen blender. The crushed leaf powder (10 g) was mixed in 100 ml of distilled water and boiled in a water bath for 30 min at 60 °C. The aqueous mixture was filtered through Whatman filter paper, grade 1, and the filtrate was stored at 4 °C.

### Synthesis of *Parthenium hysterophorus* AgNPs (PHAgNPs)

PHAgNPs were synthesized by the addition of 1 ml of PH aqueous extract to 9 ml of AgNO_3_ (1 mM). The solution pH was adjusted to pH 8 with 1 N NaOH and incubated in a water bath for 30 min at 60 °C. The AgNPs were recovered by centrifugation at 15000 rpm for 20 min, and the resulting pellet was air dried and stored in ambient conditions until further use.

### Characterization of PHAgNPs

The biosynthesized PHAgNPs were initially confirmed by UV-visible spectroscopy (Cyber lab-100 spectrophotometer), by measuring the absorbance spectra between wavelengths of 300 and 700 nm at regular intervals.

FTIR analysis was carried out to identify the reduced biomolecules from PH leaf extract in PHAgNPs. The PHAgNPs were pelletized by mixing with KBr and subjected to FTIR analysis with a JASCO 460 PLUS FTIR spectrophotometer, and the spectra were recorded between 4000 cm^−1^ and 400 cm^−1^ wavelengths.

TEM images were obtained to visualize the morphological features of PHAgNPs, with a JEOL 3010 transmission electron microscope at an acceleration voltage of 100 kV.

XRD analysis was performed to determine the structure and dimensions of PHAgNPs. The PHAgNP samples were lyophilized and subjected to XRD analysis with an XRD 600 instrument, Shimadzu, Japan instruments (voltage 40 kV; current 30 mA; CuKα radiation).

The mean particle size (L) (PAN analytical X-pert PRO Model) of the AgNPs was calculated with the following formula: L = 0.9 λ/β cos θ, per the Debye-Scherrer equation.

Where λ is the wavelength of the X-ray; θ is Bragg’s angle; and β is the full width at half maximum.

### Medium preparation

MS medium^39^ supplemented with casein hydrolysate 500 mg l^−1^, proline 500 mg l^−1^, Gamborg B5 vitamins (HiMedia, India) and sucrose 3%, was used as the rice culture medium. PHAgNPs at different concentrations (5 mg l^−1^, 10 mg l^−1^, 15 mg l^−1^ and 20 mg l^−1^) suspended in water were supplemented into the culture medium, and the pH was adjusted to pH 5.8, this was followed by sonication of the medium for 30 min. The medium was then appropriately distributed into conical flasks (50 ml medium per150 ml conical flask), and finally 4 g l^−1^ CleriGel (HiMedia, India) was added before autoclaving. After autoclaving, the medium was allowed to cool to ~45 °C. The flasks were then swirled manually and kept at 4 °C to solidify quickly, avoiding agglomeration of AgNPs.

### Influence of PHAgNPs on callus induction

*Oryza sativa* cv. IR64 seeds were obtained from TNAU, Tamil Nadu, India. The seeds were sterilized with 0.1% HgCl_2_ and 70% ethanol as previously described^40^. The sterilized IR64 seeds were inoculated in flasks containing callus induction medium (rice culture medium with 2,4-D, 2.5 mg l^−1^ and PHAgNPs) and cultured in a growth chamber at 28 ± 1 °C in the dark for 14 days. The callus induction frequency was calculated with the formula:

Callus induction frequency (%) = No. of seeds producing calli/No. of inoculated seeds

### Influence of PHAgNPs on rice plant regeneration and rhizogenesis

The embryogenic calli were transferred to regeneration medium (rice culture medium with 0.1 mg l^−1^ NAA and 3 mg l^−1^ BAP) with different concentrations of PHAgNPs (0, 5, 10, 15 and 20 mg l^−1^), and were incubated in a dark growth chamber at 28 ± 1 °C for 7 days. The flasks were then transferred to a chamber with a 16/8 h, day/night cycle. After 14 days, the regenerating calli were sub-cultured onto fresh medium with appropriate supplements every 14 days, until the plants regenerated. After 45–50 days on regeneration medium, the plant regeneration frequency was determined with the following formula:

Regeneration frequency (%) = No. of calli regenerating into plants/No. of calli initially inoculated onto regeneration medium × 100.

The regenerated plants were then transferred to rice culture medium supplemented with different concentrations of PHAgNPs (0, 5, 10, 15 and 20 mg l^−1^), and incubated in a growth chamber. After 14 days, the root length and number of roots per plant were recorded.

### Total RNA isolation and cDNA synthesis

Total RNA was isolated with a PureLink RNA Mini Kit (Ambion, USA) from 14 day old regenerating calli cultured on control and PHAgNP (5 mg l^−1^, 10 mg l^−1^, 15 mg l^−1^ and 20 mg l^−1^) supplemented medium. The isolated RNA was verified by UV absorption spectrophotometry at 260/280 nm (BioDrop duo, UK) and agarose gel electrophoresis.

cDNA was synthesized with 1 µg of RNA from individual samples with a SuperScript™ III First-Strand Synthesis Kit (Thermo Fisher Scientific, USA), according to the manufacturer’s instructions.

### qRT-PCR

The primers (Table 2) were synthesized for the auxin responsive gene *OsIAA1*^41^, cytokinin responsive gene *RR2*^42^, gibberellic acid responsive gene *PBZ1*^43^, ethylene responsive gene *ERF063*^44^ and ABA responsive gene *OsRab16*^45^ with the Primer quest tool, IDT (https://eu.idtdna.com/Primerquest/Home/Index).

**Table 2:** Quantitative RT-PCR primers.

Sl. No	Name	Forward Primer	Reverse Primer	Accession number
1	ERF063	AGCACACGCCATCAGATATT	CTCCTCCACACATCGTGATATT	CP018165.1
2	OsRab16	CACGAGTTCAGGGATCTAGCC	AGTTCTCCATCCTCTCAAGCAA	X52422.1
3	OsIAA1	CACCGCAACTGGGAACAATA	GACATCTCCAACAAGCATCCA	AJ251791
4	RR2	GGACTAGCCATGGTGATGAATG	GAGCAGGATGAGCTTGAAGATG	AJ938071.1
5	PBZ1	CCCTGCCGAATACGCCTAA	CTCAAACGCCACGAGAATTTG	D82066.1
6	UBQ5	ACCACTTCGACCGCCACTACT	ACGCCTAAGCCTGCTGGTT	AK061988

The real-time PCR reaction mixture included cDNA (1 μl), corresponding primers (0.2 μM) and 2× IQ SYBR Green Super Mix (Bio-Rad) (10 μl), adjusted to a final volume of 20 μl with sterile water.

The qRT-PCR was run on a Roche Light cycler (USA) with initial denaturation for 7 min at 95 °C, followed by 40 cycles of 95 °C for 15 s, 56 °C for 15 s, and 72 °C for 15 s; melting curve analysis was performed. Three biological replicates were analysed for each gene control and each treatment. The expression levels of each gene were normalized to the expression level of *UBQ5*^46^ on the basis of threshold cycle (Ct) values, with the 2^−ΔΔCT^ method^47^.

### Malondialdehyde content

MDA content was determined as previously described^48^ with a few modifications. One hundred milligrams of regenerating callus tissue was homogenized in 5 ml of 10% trichloroacetic acid (TCA). The homogenate was centrifuged at 10000 rpm for 30 min at room temperature. Two millilitres of supernatant was collected, and 4 µl of 0.6% thiobarbituric acid prepared in 10% TCA was added. The mixture was then incubated in a water bath at 80 °C for 40 min and immediately cooled in an ice bath and centrifuged at 10000 rpm for 30 min. The absorbance was read at 450, 532 and 600 nm, and the MDA content was calculated with the formula:

$$[{\rm{MDA}}]\,{\rm{\mu }}M\,{{\rm{g}}}^{-1}{\rm{FW}}=6.45({A}_{532}\mbox{--}{A}_{600})-0.56\,{A}_{450}$$

### Hydrogen peroxide estimation

Hydrogen peroxide was quantified as previously described^13^. One gram of callus tissue was homogenized in 10 ml of aqueous TCA (0.1%, w/v). The mixture was centrifuged at 10000 rpm for 30 min at 4 °C, and the supernatant was retrieved. One millilitre of supernatant was mixed with 4 ml of 1 M potassium iodide reagent and 1 ml of potassium phosphate buffer. The solution was incubated in the dark for 1 h at room temperature. The absorbance was read at 390 nm and plotted against a standard H_2_O_2_ curve. The results are expressed as µM g^−1^ FW.

### Total proline estimation

Proline content estimation was carried out as previously described^49^. One gram of callus tissue was homogenized in 10 ml of 5-sulphosalicylic acid (3%). The mixture was centrifuged at 10000 rpm for 15 min, and the supernatant (2 ml) was mixed well with 5 ml of glacial acetic acid and 5 ml of 140 mM acid ninhydrin, and heated for 1 h at 100 °C. The mixture was cooled and then extracted in a separating funnel with 10 ml toluene. The absorbance of the separated toluene containing the chromophore was read at 520 nm and plotted against the proline standard curve. The results are expressed as µg g^−1^ FW.

### Statistical analysis

All the experiments were carried out in triplicate (n = 3). The data were analysed in Windows Microsoft Excel 2007 software. The differences in mean values were determined with Duncan’s multiple range test, with a significance level of p ≤ 0.05.

## Data Availability

All data generated or analysed during this study are included in this published article.
